# Association of volatile substance, nitrous oxide and alkyl nitrate use with mental health in UK adolescents

**DOI:** 10.1192/bjp.2024.128

**Published:** 2025-01

**Authors:** Jemma Hawkins, Lindsey A. Hines, Chris Bonell, Matthew Hickman, Linda Adara, Julia Townson, Rebecca Cannings-John, Laurence Moore, James White

**Affiliations:** DECIPHer, School of Social Sciences, Cardiff University, UK; Department of Psychology, University of Bath, UK; Department of Public Health, Environment and Society, London School of Hygiene & Tropical Medicine, UK; Population Health Sciences, Bristol Medical School, University of Bristol, UK; Centre for Trials Research, School of Medicine, Cardiff University, UK; MRC/CSO Social and Public Health Sciences Unit, Institute of Health and Wellbeing, University of Glasgow, UK

**Keywords:** Volatile substances, nitrous oxide, alkyl nitrate, inhalants, mental health

## Abstract

**Background:**

‘Inhalants’ have been associated with poorer mental health in adolescence, but little is known of associations with specific types of inhalants.

**Aims:**

We aimed to investigate associations of using volatile substances, nitrous oxide and alkyl nitrates with mental health problems in adolescence.

**Method:**

We conducted a cross-sectional analysis using data from 13- to 14-year-old adolescents across England and Wales collected between September 2019 and March 2020. Multilevel logistic regression examined associations between lifetime use of volatile substances, nitrous oxide and alkyl nitrates with self-reported symptoms of probable depression, anxiety, conduct disorder and auditory hallucinations.

**Results:**

Of the 6672 adolescents in the study, 5.1% reported use of nitrous oxide, 4.9% volatile solvents and 0.1% alkyl nitrates. After accounting for multiple testing, adolescents who had used volatile solvents were significantly more likely to report probable depressive (odds ratio = 4.59, 95% CI 3.58, 5.88), anxiety (odds ratio = 3.47, 95% CI 2.72, 4.43) or conduct disorder (odds ratio = 7.52, 95% CI 5.80, 9.76) and auditory hallucinations (odds ratio = 5.35, 95% CI 4.00, 7.17) than those who had not. Nitrous oxide use was significantly associated with probable depression and conduct disorder but not anxiety disorder or auditory hallucinations. Alkyl nitrate use was rare and not associated with mental health outcomes. Adjustment for use of other inhalants, tobacco and alcohol resulted in marked attenuation but socioeconomic disadvantage had little effect.

**Conclusion:**

To our knowledge, this study provides the first general population evidence that volatile solvents and nitrous oxide are associated with probable mental health disorders in adolescence. These findings require replication, ideally with prospective designs.

## Relevance statement

Inhalants are commercial products or chemicals that people intentionally inhale vapours from to achieve intoxication. There are three main types: volatile solvents (e.g. glues, solvents, petrol), nitrous oxide and alkyl nitrates (i.e. ‘poppers’, including chemicals such as amyl nitrite, pentyl nitrite and butyl nitrite). These inhalants have different pharmacological properties, effects and contexts of use, yet they are often grouped together.^1^ This grouping is a significant barrier in estimating prevalence, characterising users and estimating associations with health outcomes. For example, while all of these substances have intoxicating effects, alkyl nitrates have muscle-relaxing physiological effects and thus their use is common in a sexual context as well as for intoxication purposes.^1^ The available data on lifetime prevalence among adolescents suggest the following range in use between regions: 18% use of ‘volatile solvents’ in Australia (7% past month, 4% past week, 12–17 years old),^2^ 2.6% use of volatile substances and nitrous oxide in the USA (1.9% in past 30 days, 13–14 years old)^3^ and 7.2% in Europe (lifetime use, 15–16 years old).^4^ The European survey allowed countries to use different questions so it is unclear which inhalants were included. The only prevalence data in adolescents we could find that reported on each type of inhalant separately was from 11- to 15-year-olds in England, where 6.8% reported use of volatile substances (2.4% past month), 3.0% nitrous oxide (2.0% past month) and 0.6% (0.7% past month) alkyl nitrates.^5^

### Inhalant use and adolescent mental health

Studies examining the association between combined inhalant use and mental health outcomes in adolescents have reported mixed results. In two convenience samples with US incarcerated youth,^6,7^ inhalant use was associated with anxiety and depressive symptoms.^6^ In the two prospective studies, inhalant use in 125 American Indian youth was associated with symptoms of conduct disorder 4 years later,^8^ and in the largest study to date, the Northern Finland Birth Cohort (*n* = 6542), a dose–response relationship was reported between inhalant use at 16 years old and an increased risk of psychosis at 30 years old, after adjusting for alcohol, tobacco and other illicit drug use.^9^

### Volatile solvents, nitrous oxide and alkyl nitrate use and adolescent mental health

Few studies have examined separate associations between volatile solvents, nitrous oxide and alkyl nitrates and mental health outcomes. In the four cross-sectional studies investigating volatile solvents, use was associated with suicide, depressive symptoms, weapon carrying and fighting in 354 14- to 20-year-olds attending a US alternative school (aiming to prevent school dropout),^10^ symptoms of depression, anxiety and psychoticism in a study of 723 US incarcerated youth (average age 15.5 years),^11^ psychological distress in 47 15-year-olds in the UK criminal justice system^12^ and depressive and conduct problems (with weaker evidence for anxiety disorders) in 124 students aged 12–17 years in one north-western Russian city.^13^ In the one study with US incarcerated youth (*n* = 723), lifetime nitrous oxide and volatile substance use was associated with symptoms of depression, anxiety and psychoticism, compared to those who had never used, but there was little evidence of associations with outcomes for young people who only used nitrous oxide.^11^ We could not find any studies on alkyl nitrate use and specific mental health symptoms but the US 2000–2001 National Household Surveys on Drug Abuse found 12- to 17-year-old lifetime users of alkyl nitrates were more likely than non-users to report having used mental health services in the past year.^14^

### Methodological limitations of previous research

Nearly all research to date has focused on the association between mental health problems and ‘inhalants’, with very few studies separating out specific associations between mental health problems and use of volatile substances, nitrous oxide and alkyl nitrates. Existing studies have also tended to be either small (thus likely lacking in statistical power), used convenience samples with incarcerated adolescents (which might introduce sampling bias)^15^ and not analysed volatile substance, nitrous oxide and alkyl nitrates use in the same sample (increasing concerns about specific effects and confounding). As far as we are aware, there have been no studies that have compared the association between volatile solvent, nitrous oxide and alkyl nitrate use with the risk of probable mental health disorders in a general adolescent population sample.

### Aims

To address these limitations, we aimed to: (a) describe the prevalence of nitrous oxide, volatile solvents and alkyl nitrates use among a population of UK adolescents; (b) describe the characteristics of participants reporting nitrous oxide, volatile solvents and alkyl nitrates use; (c) explore associations between the use of nitrous oxide, volatile solvents and alkyl nitrates with symptoms from a range of mental health disorders.

## Method

### Study population

These data arise from baseline assessments within a randomised controlled trial in the west of England and south Wales between September 2019 and March 2020. Secondary schools (excluding private schools, special schools, pupil referral units and schools that received the trial intervention during piloting) were randomly sampled and questionnaires self-completed before allocation. For this reason, the sample cannot be considered nationally representative; further details of the recruitment and sampling strategy are available elsewhere.^16^ Of the 311 schools within the study areas, 244 (78.5%) met the eligibility criteria. A stratified random sample of these eligible schools was invited to participate. Stratification variables were the percentage of students entitled to free school meals, an index of parental socioeconomic disadvantage (above versus below the median of schools recruited) and country (England versus Wales). Questionnaires were completed by year-nine students (aged 13–14 years) in schools, with the assistance of fieldworkers. Data were collected before random allocation of schools. The authors assert that all procedures contributing to this work comply with the ethical standards of the relevant national and institutional committees on human experimentation and with the Helsinki Declaration of 1975, as revised in 2008. Ethical approval for the study was obtained from Cardiff University's School of Social Sciences Ethics Committee (SREC/3342). An opt-out consent process was used with parents/carers and students provided written opt-in consent.

This manuscript adheres to the STROBE reporting guidelines (see Supplementary material available at https://doi.org/10.1192/bjp.2024.128).^17^

### Exposure

Participants were asked whether they have ever tried ‘Nitrous Oxide (also called: laughing gas, balloons, hippie crack). Please don't include any times you were offered it by a dentist or doctor’, volatile solvents described as ‘Inhaling or sniffing glues, gases, aerosols to get “high” (includes lighter refills, solvents, petrol, butane)’, and alkyl nitrates described as ‘Poppers (also called: amyl nitrite, liquid gold)’, with the response options of ‘Yes I have tried’ and ‘No I have not tried’.

### Outcomes

Participants were asked to complete scales that assessed symptoms of three mental health disorders and auditory hallucinations. For each scale, we indicate the ‘probable’ presence of a disorder where a validated cut-off was exceeded.

#### Probable depressive disorder

Participants completed the 13-item Short Mood and Feelings Questionnaire, which assesses depressive symptoms in children and adolescents over the past 2 weeks.^18^ We applied the ≥12 cut-off point to indicate a disorder.^18^

#### Probable anxiety disorder

Participants completed the seven-item Generalised Anxiety Disorder (GAD-7) scale, which assesses anxiety symptoms over the past 2 weeks.^19^ We applied the ≥10 cut-off point considered indicative of a disorder.^19^

#### Auditory hallucinations

Auditory hallucinations were self-reported using questions from the World Health Organization Composite International Diagnostic Interview.^20^

#### Probable conduct disorder

The six-item Oregon Adolescent Depression Project Conduct Disorder Screener was completed by participants.^21^ We applied the ≥9 cut-off point as indicative of a disorder.^21^

#### Covariates

Gender identity was self-reported and categorised as boy, girl or a gender minority (transboy, transgirl, non-binary (neither male or female), unsure/questioning, other, prefer not to say). Participants also reported their age (years), ethnicity (‘Black and minority ethnic’, comprising: ‘Asian or Asian British’, ‘Black or Black British’ and ‘Mixed/multiple ethnic backgrounds’, and ‘White’, comprising: ‘White British’ and ‘White not British’), socioeconomic disadvantage (whether any adults they lived with were employed, a single-item on free-school-meal entitlement^22,23^) weekly smoking status (at least one cigarette a week) and consumption of a whole alcoholic drink in the past 30 days.

### Statistical analysis

To describe the young people who reported using nitrous oxide, volatile solvents and alkyl nitrates, we compared the demographic characteristics, socioeconomic disadvantage, weekly smoking status and the use of alcohol in the past 30 days to those not using these substances, using separate multilevel univariable logistic regression models. The associations between use of nitrous oxide, volatile solvents and alkyl nitrates and mental health outcomes were analysed using univariable and multivariable multilevel logistic regression models (students nested within schools). Three separate multivariable logistic regression models were performed for the association between the reported use with each outcome, compared to never having used, to examine the potential confounding effects of: (a) adjustment for the other inhalants (e.g. for nitrous oxide, we adjusted for use of volatile solvents and alkyl nitrates), (b) adjusted as in model 1, with inclusion of gender identity, age, ethnicity and socioeconomic disadvantage, and (c) adjusted as in model 2 with tobacco and alcohol use. All results are presented as odds ratios with 95% CIs. To reduce the risk of generating spurious findings due to multiple testing, the threshold for significance for the comparison was Bonferroni-adjusted to *P* < 0.001 (*P* = 0.05/48). Sensitivity analyses were conducted after excluding participants with any missing data and applying inverse-probability weighting by a propensity score (Supplementary Tables 2 and 3). Analyses were performed in Stata version 17.0 (Stata Corp LLC, College Station, Texas; see https://www.stata.com/stata17/). The analyses were not pre-registered and therefore the results should be considered exploratory.

#### Missing data and imputation

Missing data per variable ranged from 1.0 to 11.4% (Supplementary Table 1). Missing data in all variables (exposures, outcomes, and covariates) were addressed through multiple imputation using chained equations including all variables as predictors. Estimates were obtained by pooling results across 20 imputed data-sets, using the Rubin rules, and assessment of Monte Carlo errors suggested this was a suitable number of imputations.^24^

## Results

Of 7077 eligible year-9 students (aged 13–14 years), 6672 participated (94.3% response) with 5.1% reporting use of nitrous oxide, 4.9% volatile solvents and 0.1% alkyl nitrates. Use of nitrous oxide was more common in participants who were Black and minority ethnic (7.0 *v.* 4.8%), were entitled to free school meals (7.8 *v.* 4.6%), lived with no adults in employment (10.3 *v.* 4.8%), were weekly cigarette smokers (35.9 *v.* 4.6%) and had consumed alcohol in the past 30 days (9.0 *v.* 2.6%). Use of volatile solvents was more common in those who identified as a gender minority or girl (13.4 *v.* 5.2 (girl) and 4.2% (boy)), were entitled to free school meals (7.4 *v.* 4.2%), lived with no employed adults (8.9 *v.* 4.6%), were weekly cigarette smokers (42.9 *v.* 4.3%) and had consumed alcohol in the past 30 days (10.6 *v.* 1.3%). The characteristics of alkyl nitrate users were very similar to those for volatile solvents (Table 1).

**Table 1:** Association between lifetime nitrous oxide, volatile solvent and alkyl nitrate use and participant characteristics (*n* = 6672)

	Nitrous oxide, %			Volatile solvent, %			Alkyl nitrates, %		
Characteristic	Never (*n* = 6332)^a^	Used (*n* = 340)	*P*-value^b^	Never (*n* = 6345)	Used (*n* = 327)	*P*-value^b^	Never (*n* = 6645)	Used (*n* = 27)	*P*-value^b^
Gender identity									
Boy	95.0	5.0		95.8	4.2		99.7	0.3	
Girl	95.1	4.9	0.86	94.8	5.2	0.05	99.6	0.4	0.64
Gender Minority^c^	92.2	7.8	0.16	86.6	13.4	<0.001	97.6	2.4	0.002
Ethnicity									
White	95.2	4.8		95.3	4.7		99.7	0.3	
Black and minority ethnic	93.0	7.0	0.005	93.8	6.2	0.07	98.4	1.4	0.003
Entitled to free school meals									
Yes	93.0	7.0		92.6	7.4		99.1	0.9	
No	95.4	4.6	0.01	95.8	4.2	<0.001	99.7	0.3	0.02
Don't know	92.0	8.0	0.001	92.0	8.0	<0.001	99.1	0.9	0.02
Parent(s) unemployment									
Unemployed	89.7	10.3		91.1	8.9		99.0	1.0	
Employed	95.2	4.8	<0.001	95.4	4.6	<0.001	99.6	0.4	0.10
Weekly cigarette smoking									
Not smoked	95.4	4.6		95.7	4.3		99.7	0.3	
Smoked	64.1	35.9	<0.001	57.1	42.9	<0.001	90.5	9.5	<0.001
Alcohol in past 30 days									
Not consumed	97.4	2.6		98.7	1.3		99.9	0.1	
Consumed	91.0	9.0	<0.001	89.4	10.6	<0.001	99.1	0.9	<0.001

a. All numbers estimated from imputed proportions.

b. Determined by logistic regression.

c. Gender minority comprised: transboy, transgirl, non-binary (neither male or female), unsure/questioning and other.

The prevalence of probable depressive disorder was 22.6% (95% CI, 21.6–23.7), anxiety disorder 19.9% (95% CI, 18.9–20.9), auditory hallucination 17.5% (95% CI, 16.5–18.6) and conduct disorder 22.1% (95% CI, 21.1–23.2). There was a significant unadjusted association between the use of nitrous oxide with probable depressive disorder (OR, 2.87; 95% CI, 2.27–3.63), anxiety disorder (2.57; 95% CI, 2.03–3.27), auditory hallucinations (5.18; 95% CI, 4.00–6.69) and conduct disorder (2.97; 95% CI, 2.21–3.98) (Table 2). There was also a significant unadjusted association between the use of volatile solvents with probable depressive disorder (OR, 4.59; 95% CI, 3.58–5.88), anxiety disorder (3.47; 95% CI, 2.72–4.43), auditory hallucination (5.35; 95% CI, 4.00–7.17) and conduct disorder (7.52; 95% CI, 5.80–9.76). Those who reported using alkyl nitrates reported symptoms consistent with probable anxiety disorder (7.79; 95% CI, 3.20–18.97), auditory hallucinations (14.29; 95% CI, 4.50–45.33) and conduct disorder (17.95; 95% CI, 6.08–53.04) with weaker evidence of an association with depressive symptoms (3.20; 95% CI, 1.38–7.42). Mutual adjustment which included all three inhalants markedly reduced associations, with those for alkyl nitrates virtually eliminated. There was little evidence of attenuation of these associations after adjustment for sociodemographic factors. Associations were further reduced after additional adjustment for tobacco and alcohol use. Nitrous oxide remained significantly associated with probable depressive and conduct disorder but not with probable anxiety or auditory hallucinations. Participants reporting use of volatile substances remained around 2–3 times more likely to report a probable disorder or an auditory hallucination compared to their peers who reported no use (Table 2).

**Table 2:** Odds ratio (95% CI) for association between nitrous oxide, volatile solvents and alkyl nitrates with probable depressive disorder, generalised anxiety disorder, conduct disorder and auditory hallucinations (*n* = 6672)^a^

Lifetime exposure	Unadjusted	Adjustment for other exposures (model 1)	Adjusted for sociodemographic factors (model 2)^b^	Adjusted for tobacco and alcohol use (model 3)^c^
	Probable depressive disorder			
Nitrous oxide	2.87 (2.27, 3.63)***	1.96 (1.51, 2.54)***	2.18 (1.66, 2.87)***	1.77 (1.33, 2.38)***
Volatile solvents	4.59 (3.58, 5.88)***	3.81 (2.92, 4.97)***	3.79 (2.86, 5.02)***	2.55 (1.90, 3.42)***
Alkyl nitrates	3.20 (1.38, 7.42)	0.86 (0.34, 2.16)	0.70 (0.26, 1.88)	0.48 (0.18, 1.34)
	Probable generalised anxiety disorder			
Nitrous oxide	2.57 (2.03, 3.27)***	1.77 (1.36, 2.31)***	1.91 (1.44, 2.52)***	1.59 (1.19, 2.52)
Volatile solvents	3.47 (2.72, 4.43)***	2.76 (2.12, 3.59)***	2.60 (1.96, 3.43)***	1.83 (1.37, 2.45)***
Alkyl nitrates	7.79 (3.20, 18.97)***	2.91 (1.14, 7.44)	2.74 (1.00, 7.52)	2.10 (0.74, 5.90)
	Auditory hallucination^d^			
Nitrous oxide	2.97 (2.21, 3.98)***	1.81 (1.30, 2.52)***	1.76 (1.26, 2.47)***	1.50 (1.07, 2.11)
Volatile solvents	5.35 (4.00, 7.17)***	4.23 (3.10, 5.79)***	3.82 (2.77, 5.25)***	2.68 (1.92, 3.73)***
Alkyl nitrates	14.29 (4.50, 45.33)***	4.02 (1.19, 13.60)	3.58 (1.01, 12.58)	2.79 (0.80, 9.78)
	Conduct disorder			
Nitrous oxide	5.18 (4.00, 6.69)***	3.28 (2.47, 4.36)***	3.23 (2.43, 4.30)***	2.51 (1.85, 3.40)***
Volatile solvents	7.52 (5.80, 9.76)***	5.35 (4.06, 7.05)***	5.28 (4.00, 6.98)***	3.14 (2.33, 4.25)***
Alkyl nitrates	17.95 (6.08, 53.04)***	3.99 (1.20, 13.31)	3.74 (1.10, 12.81)	2.06 (0.62, 6.79)

a. All results estimated from imputed data. Multivariable model adjustment is incremental and additive (i.e. model 3 includes the variables from models 1 and 2 plus the tobacco and alcohol variables).

b. Sociodemographic factors comprised: gender identity, ethnicity, free-school-meal entitlement and living with an employed parent.

c. Tobacco use assessed as weekly cigarette smoking and alcohol assessed as consuming alcohol in the past 30 days.

d. Analytical *n* = 4813 as excludes students who responded that they preferred not to say or didn't know whether they had hallucinated.

*** Statistically significant under a Bonferroni-corrected *P* < 0.001 for 48 comparisons.

In the data-sets where there were no missing data, the CIs for estimates overlapped with those from the main results using imputed data, indicating there were no meaningful differences (Supplementary Table 2). There was little difference between the estimates from the multivariable and propensity-score model (Supplementary Table 3).

## Discussion

### Main findings

In a general population sample of 13- to 14-year-olds in the UK we found a strong association between individuals using nitrous oxide and volatile solvents and mental health symptoms consistent with a probable depressive disorder, anxiety disorder, auditory hallucinations and conduct disorder. Alkyl nitrate exposure was rare, at 0.1%. The associations for nitrous oxide and alkyl nitrates were largely explained by differences in the use of other inhalants and tobacco and alcohol use. In contrast, use of volatile substances was consistently associated with all probable mental health disorders even after adjusting for these factors. To our knowledge, the present study gives the first user profiles for nitrous oxide, volatile solvents and alkyl nitrate users in early adolescence, indicating that use was more common among young people who report being a weekly cigarette smoker, have used alcohol in the past 30 days or who live with unemployed parents or with a relatively low income. Use of volatile substances and alkyl nitrate, but not nitrous oxide, was more common in young people identifying as a gender minority than boy or girl.

### Comparison with previous findings

Our findings replicate the demographic patterning from previous studies that report on a combined exposure of inhalants in that we found users were more likely to have a lower household income,^8,25^ report alcohol and tobacco use,^7,8,10^ but our findings extend this research to show specific patterns of use of volatile substances and alkyl nitrate use according to gender identity. We found alkyl nitrate use was around six times more common in young people identifying as a gender minority than boy or girl. This pattern of use may be related to gender minority young people also being more likely to identify as homosexual or bisexual,^26^ as there is well documented higher prevalence of alkyl nitrates use among men who have sex with men than heterosexual men.^27^ Given the age of the population studied being below the age of consent, future research is required to further understand this pattern of use and inform harm-reduction practices.

Volatile solvent use was associated with all the mental health disorders assessed. Four small US^10,11^ and UK^12^ cross-sectional studies with incarcerated youth and one general population Russian study^13^ found volatile solvent users were more likely to report symptoms of anxiety and depression.^7,10,12^ The present results extend this literature to demonstrate an association between volatile substances and negative mental health outcomes at an earlier age than has previously been shown and in a general population sample. Analyses did not show attenuation of these associations after adjustment for socioeconomic disadvantage. This contrasts previous studies showing higher use in American Indian youths living in reserves with poor access to schooling compared to American Indians living in other areas with better access.^28^

Nitrous oxide was associated with a significant increased risk of reported symptoms of probable depression and conduct disorder but not anxiety disorder or auditory hallucinations. In an international study of nitrous oxide use with a self-selecting general population sample,^29^ hallucinations were the most commonly reported adverse effect, with 27.8% of the sample reporting experiencing these in the past 12 months. However, the study did not report on an adolescent sample nor provide further information on types of hallucinations. One small convenience sample of US incarcerated youth (*n* = 723) reported an unadjusted association between lifetime nitrous oxide and volatile substance use and symptoms of depression, anxiety and psychoticism compared to those who had never used, but little evidence of an association with mental health outcomes in the group that only used nitrous oxide.^11^ This study did, however, include 87% males, with 42.3% currently prescribed psychotropic medication, and only 4% (*n* = 11) of participants reported sole nitrous oxide use, suggesting analyses were likely to be underpowered with limited generalisability to the wider general population of young people.

Alkyl nitrate use was not associated with mental health outcomes in our analysis, with only 0.1% of students reporting using alkyl nitrates. The only other estimates of prevalence in the literature come from the 2000–2001 National Household Surveys on Drug Abuse,^14^ in which 1.5% of 12- to 17-year-olds reported lifetime use. Aside from reviews of case reports of poppers maculopathy^30^ and methemoglobinemia^27^ associated with chronic use, there is very little research on alkyl nitrate use and physical or mental health outcomes. We could find no prior research on alkyl nitrates and mental health outcomes in adolescents.

### Limitations

This study has some limitations. First, the cross-sectional design of the study means we cannot establish whether the associations reported are causal, or rule out reverse causality. It is possible that young people with existing mental health problems choose to use volatile solvents or nitrous oxide as a form of self-medication. It is conceivable that young people may do this, as emerging evidence indicates nitrous oxide may be an effective treatment for depressive disorders.^31^  Second, we cannot be certain that participants are correctly reporting the type of inhalant they used. We have attempted to mitigate this limitation by expanding descriptions of each inhalant and including street names. Third, an alternative to a causal explanation is that these associations are brought about by confounding, whereby both volatile substance use and probable mental health disorder share common antecedents such as exposure to adverse childhood experiences.^32^ Fourth, alkyl nitrates had low prevalence, and associations with outcomes had wide CIs, indicating there is a need for much larger studies. Finally, while analyses showed that use of all substances was more common among those who reported weekly cigarette smoking; the question used to measure this did not explicitly include or exclude e-cigarette use.

### Implications

Using data from a general population sample in the UK, we found that reports of volatile substance use were associated with an increased risk of three probable mental health disorders and auditory hallucinations. General population studies are useful in estimating associations at the population level and can be crucial for informing policy-makers and clinical service providers. Our findings signal to clinicians, educators and policy-makers that there is a need for education on the use of volatile substances among young people as a marker of an increased risk of mental health problems. Given volatile substances are one of the most frequently used illicit drugs in adolescence, these results provide psychiatrists with evidence for informed discussions with people about their use of these substances. Further prospective evidence is required, ideally with large samples, into the effects of specific inhalants on probable mental health disorders and exploration of the impact of chronic use as well as lifetime use.

## Supporting information

Hawkins et al. supplementary material Hawkins et al. supplementary material

## Data Availability

The data that support the findings of this study are openly available at http://doi.org/10.17035/d.2023.0244798057

## References

[1] Crossin R, Whelan J, Ball J. Defining and measuring ‘inhalant’ use in population-based surveys. Int J Drug Policy [Epub ahead of print] 3 Mar 2023. Available from: 10.1016/j.drugpo.2023.103991. 36870868

[2] Australian Government Department of Health and Aged Care. Secondary School Students’ Use of Tobacco, Alcohol and Other Drugs in 2017. Australian Government Department of Health and Aged Care, 2019 (https://www.health.gov.au/resources/publications/secondary-school-students-use-of-tobacco-alcohol-and-other-drugs-in-2017?language=en).

[3] Miech RA, Johnston LD, O'Malley PM, Bachman JG, Schulenberg JE, Patrick ME. Monitoring the Future National Survey Results on Drug Use, 1975–2021: Volume 1, Secondary School Students. Institute for Social Research, 2022 (https://eric.ed.gov/?id=ED619855).

[4] European Monitoring Centre for Drugs and Drug Addiction. ESPAD Report 2019: Results From the European School Survey Project on Alcohol and Other Drugs. Publications Office, 2020 (https://data.europa.eu/doi/10.2810/877033).

[5] NHS Digital. Smoking, Drinking and Drug Use among Young People in England, 2021: Data Tables. NHS Digital, 2022 (https://digital.nhs.uk/data-and-information/publications/statistical/smoking-drinking-and-drug-use-among-young-people-in-england/2021/data-tables).

[6] McGarvey EL, Canterbury RJ, Waite D. Delinquency and family problems in incarcerated adolescents with and without a history of inhalant use. Addict Behav 1996; 21(4): 537–42. 8830913 10.1016/0306-4603(95)00074-7

[7] Howard MO, Balster RL, Cottler LB, Wu LT, Vaughn MG. Inhalant use among incarcerated adolescents in the United States: prevalence, characteristics, and correlates of use. Drug Alcohol Depend 2008; 93(3): 197–209. 17983710 10.1016/j.drugalcdep.2007.08.023

[8] Howard MO, Walker RD, Walker PS, Cottler LB, Compton WM. Inhalant use among urban American Indian youth. Addiction 1999; 94(1): 83–95. 10665100 10.1046/j.1360-0443.1999.941835.x

[9] Mustonen A, Niemelä S, McGrath JJ, Murray GK, Nordström T, Mäki P, et al. Adolescent inhalant use and psychosis risk – a prospective longitudinal study. Schizophr Res 2018; 201: 360–6. 29958751 10.1016/j.schres.2018.05.013

[10] Fleschler MA, Tortolero SR, Baumler ER, Vernon SW, Weller NF. Lifetime inhalant Use among alternative high school students in Texas: prevalence and characteristics of users. Am J Drug Alcohol Abuse 2002; 28(3): 477–95. 12211361 10.1081/ada-120006737

[11] Garland EL, Howard MO, Perron BE. Nitrous oxide inhalation among adolescents: prevalence, correlates, and co-occurrence with volatile solvent inhalation. J Psychoact Drugs 2009; 41(4): 337–47. 10.1080/02791072.2009.10399771 PMC2921531 20235440

[12] Jacobs AM, Ghodse AH. Delinquency and regular solvent abuse: an unfavourable combination? Br J Addict 1988; 83(8): 965–8. 3167252 10.1111/j.1360-0443.1988.tb01590.x

[13] Koposov R, Stickley A, Ruchkin V. Inhalant use in adolescents in northern Russia. Soc Psychiatry Psychiatr Epidemiol 2018; 53(7): 709–16. 29721591 10.1007/s00127-018-1524-z PMC6003974

[14] Wu LT, Schlenger WE, Ringwalt CL. Use of nitrite inhalants (“poppers”) among American youth. J Adolesc Health 2005; 37(1): 52–60. 15963907 10.1016/j.jadohealth.2004.06.007

[15] Kurtzman TL, Otsuka KN, Wahl RA. Inhalant abuse by adolescents. J Adolesc Health 2001; 28(3): 170–80. 11226839 10.1016/s1054-139x(00)00159-2

[16] White J. A Multicentre Cluster Randomised Controlled Trial to Evaluate the Effectiveness and Cost-effectiveness of a School-based Peer-led Drug Prevention Intervention (The FRANK Friends Study). National Institute of Health Research (NIHR), 2019 (https://www.journalslibrary.nihr.ac.uk/programmes/phr/179702/#/).

[17] von Elm E, Altman DG, Egger M, Pocock SJ, Gøtzsche PC, Vandenbroucke JP. STrengthening the Reporting of OBservational Studies in Epidemiology (STROBE) statement: guidelines for reporting observational studies. Br Med J 2007; 335(7624): 806–8. 17947786 10.1136/bmj.39335.541782.AD PMC2034723

[18] Ancold A, Stephen C. Development of a short questionnaire for use in epidemiological studies of depression in children and adolescents. Int J Methods Psychiatr Res 1995; 5(4): 237–49.

[19] Spitzer RL, Kroenke K, Williams JBW, Löwe B. A brief measure for assessing generalized anxiety disorder: the GAD-7. Arch Intern Med 2006; 166(10): 1092–7. 16717171 10.1001/archinte.166.10.1092

[20] Kessler RC, Üstün TB. The world mental health (WMH) survey initiative version of the World Health Organization (WHO) composite international diagnostic interview (CIDI). Int J Methods Psychiatr Res 2004; 13(2): 93–121. 15297906 10.1002/mpr.168 PMC6878592

[21] Lewinsohn PM, Rohde P, Farrington DP. The OADP-CDS: a brief screener for adolescent conduct disorder. J Am Acad Child Adolesc Psychiatry 2000; 39(7): 888–95. 10892231 10.1097/00004583-200007000-00018

[22] Ilie S, Sutherland A, Vignoles A. Revisiting free school meal eligibility as a proxy for pupil socio-economic deprivation. Br Educ Res J 2017; 43(2): 253–74.

[23] Taylor C. The reliability of free school meal eligibility as a measure of socio-economic disadvantage: evidence from the millennium cohort study in Wales. Br J Educ Stud 2018; 66(1): 29–51.

[24] White IR, Royston P, Wood AM. Multiple imputation using chained equations: issues and guidance for practice. Stat Med 2011; 30(4): 377–99. 21225900 10.1002/sim.4067

[25] Nakawaki B, Crano W. Patterns of substance use, delinquency, and risk factors among adolescent inhalant users. Subst Use Misuse 2015; 50(1): 114–22. 25290663 10.3109/10826084.2014.961611 PMC4687965

[26] White J, Trinh MH, Reynolds CA. Psychological distress, self-harm and suicide attempts in gender minority compared with cisgender adolescents in the UK. BJPsych Open 2023; 9(5): e138. 37525614 10.1192/bjo.2023.534 PMC10486222

[27] Romanelli F, Smith KM, Thornton AC, Pomeroy C. Poppers: epidemiology and clinical management of inhaled nitrite abuse. Pharmacotherapy 2004; 24(1): 69–78. 14740789 10.1592/phco.24.1.69.34801

[28] National Inhalant Abuse Taskforce. National Directions on Inhalant Abuse: Consultation Paper. National Inhalant Abuse Taskforce, 2005.

[29] Kaar SJ, Ferris J, Waldron J, Devaney M, Ramsey J, Winstock AR. Up: the rise of nitrous oxide abuse. An international survey of contemporary nitrous oxide use. J Psychopharmacol 2016; 30(4): 395–401. 26912510 10.1177/0269881116632375

[30] Bartolo C, Koklanis K, Vukicevic M. ‘Poppers maculopathy’ and the adverse ophthalmic outcomes from the recreational use of alkyl nitrate inhalants: a systematic review. Semin Ophthalmol 2023; 38(4): 371–9. 35938499 10.1080/08820538.2022.2108717

[31] Rech P, Custodio RM, Rodrigues Uggioni ML, Silveira Prestes G, Marçal F, Silveira VP, et al. Use of nitrous oxide in the treatment of major depressive disorder and treatment-resistant major depressive disorder: a systematic review and meta-analysis nitrous oxide in depressive disorders. Prog Neuro-Psychopharmacol Biol Psychiatry 2024; 129: 110869. 10.1016/j.pnpbp.2023.110869 37813146

[32] Brockie TN, Dana-Sacco G, Wallen GR, Wilcox HC, Campbell JC. The relationship of adverse childhood experiences to PTSD, depression, poly-drug use and suicide attempt in reservation-based native American adolescents and young adults. Am J Community Psychol 2015; 55(3): 411–21. 25893815 10.1007/s10464-015-9721-3

