# Supplementary material for: Association of volatile substance, nitrous oxide and alkyl nitrate use with mental health in UK adolescents
Source: Br J Psychiatry. 2025 Jan;226(1):10–5. doi: 10.1192/bjp.2024.128 (PMC7617277; doi:10.1192/bjp.2024.128)
Supplement: Hawkins et al. supplementary material [file S0007125024001284sup001.docx]

**Supplementary Online Content**

**Table S1.** Number and percentage of missing data for each variable

**Table S2.** Odds ratio (95% confidence interval) for association between nitrous oxide, volatile solvents and alkyl nitrates and probable depressive disorder, generalised anxiety disorder, auditory hallucinations and conduct disorder in complete case sample (n = 3163)

**Table S3.** Odds ratio (95% confidence interval) for association between nitrous oxide, volatile solvents and alkyl nitrates with study outcomes, comparing multivariable adjustment and inverse probability weighting by a propensity score (n = 6612)

**Table S1. Number and percentage of missing data for each variable**

| **Characteristic** | **Missing** | |
| --- | --- | --- |
|  | **N** | **%** |
| *Exposure* |  |  |
| Nitrous oxide | 156 | 2.3 |
| Volatile solvents | 154 | 2.3 |
| Alkyl nitrates | 179 | 2.7 |
| *Covariates* |  |  |
| Gender identity | 68 | 1.0 |
| Ethnicity | 88 | 1.3 |
| Free school meals entitlement | 81 | 1.2 |
| Parent(s) / carer(s) unemployment | 606 | 9.1 |
| Weekly smoking status | 551 | 8.3 |
| Alcohol consumed in past 30 days | 760 | 11.4 |
| *Outcomes* |  |  |
| Probable depressive disorder | 496 | 7.4 |
| Probable anxiety disorder | 602 | 9.0 |
| Auditory hallucinations | 292 | 4.4 |
| Probable conduct disorder | 631 | 9.5 |

**Table S2. Odds ratio (95% confidence interval) for association between nitrous oxide, volatile solvents and alkyl nitrates with study outcomes in the complete case sample (n = 3163)** ^a^

|  | **Probable depressive disorder** | | | |
| --- | --- | --- | --- | --- |
| **Exposure variable** | **Unadjusted** | **Adjustment for other inhalants** | **Additionally adjusted for sociodemographic factors** ^a^ | **Adjusted for tobacco and alcohol use** ^c^ |
| **Nitrous oxide** | 4.37 (3.04, 6.28) | 2.96 (1.99, 4.40) | 3.22 (2.13, 4.87) | 2.47 (1.61, 3.79) |
| **Volatile solvents** | 4.73 (3.35, 6.68) | 3.51 (2.42, 5.08) | 3.11 (2.10, 4.62) | 1.94 (1.29, 2.94) |
| **Alkyl nitrates** | 4.09 (1.29, 12.96) | 0.96 (0.27, 3.38) | 0.88 (0.23, 3.39) | 0.44 (0.11, 1.76) |
|  |  |  |  |  |
|  | **Probable generalized anxiety disorder** | | | |
| **Nitrous oxide** | 3.67 (2.54, 5.31) | 2.40 (1.60, 3.61) | 2.49 (1.63, 3.82) | 2.02 (1.31, 3.11) |
| **Volatile solvents** | 4.24 (3.00, 5.99) | 3.17 (2.18, 4.61) | 2.78 (1.87, 4.14) | 1.92 (1.26, 2.90) |
| **Alkyl nitrates** | 6.48 (2.01, 20.88) | 1.93 (0.55, 6.78) | 1.86 (0.48, 7.18) | 1.25 (0.32, 4.86) |
|  |  |  |  |  |
|  | **Auditory hallucination** | | | |
| **Nitrous oxide** | 3.74 (2.59, 5.41) | 2.03 (1.33, 3.09) | 1.98 (1.28, 3.02) | 1.57 (1.02, 2.43) |
| **Volatile solvents** | 6.35 (4.49, 8.97) | 4.89 (3.37, 7.08) | 4.32 (2.95, 6.33) | 2.95 (1.98, 4.39) |
| **Alkyl nitrates** | 15.39 (4.09, 57.90) | 4.46 (1.06, 18.73) | 4.02 (0.91, 17.75) | 2.56 (0.58, 11.24) |
|  |  |  |  |  |
|  | **Conduct disorder** | | | |
| **Nitrous oxide** | 5.21 (3.62, 7.49) | 2.94 (1.96, 4.41) | 2.94 (1.95, 4.43) | 2.05 (1.32, 3.17) |
| **Volatile solvents** | 7.45 (5.24, 10.58) | 5.27 (3.63, 7.66) | 5.06 (3.46, 7.39) | 2.72 (1.81, 4.09) |
| **Alkyl nitrates** | 48.45 (6.17, 380.48) | 14.79 (1.65, 132.40) | 15.52 (1.69, 142.48) | 5.88 (0.64, 54.35) |

^a^ Multivariable model adjustment is incremental.

^b^ Sociodemographic factors comprised: gender identity, ethnicity, free school meal entitlement and living with an employed parent.

^c^ Tobacco use assessed as weekly cigarette smoking and alcohol assessed as consuming alcohol in the past 30 days.

^d^ Analytical n = 4813 as excludes students who responded that they preferred not to say or didn’t know whether they had hallucinated

**Table S3. Odds ratio (95% confidence interval) for association between nitrous oxide, volatile solvents and alkyl nitrates with study outcomes, comparing multivariable adjustment and inverse probability weighting by a propensity score (n = 6672) ^a^**

|  | **Probable depressive disorder** | |
| --- | --- | --- |
|  | **Multivariable adjustment ^b^** | **Inverse probability weighting ^c^**  **by propensity score ^c^** |
| **Nitrous oxide** | 1.77 (1.33, 2.38) *** | 1.03 (0.95, 1.12) |
| **Volatile solvents** | 2.55 (1.90, 3.42) *** | 1.15 (1.06, 1.25) *** |
| **Alkyl nitrates** | 0.48 (0.18, 1.34) | 0.88 (0.70, 1.10) |
|  |  |  |
|  | **Probable generalized anxiety disorder** | |
| **Nitrous oxide** | 1.59 (1.19, 2.52) | 1.01 (0.93, 1.10) |
| **Volatile solvents** | 1.83 (1.37, 2.45) *** | 1.08 (1.00, 1.16) |
| **Alkyl nitrates** | 2.10 (0.74, 5.90) | 1.21 (0.96, 1.51) |
|  |  | |
|  | **Auditory hallucinations** ^d^ | |
| **Nitrous oxide** | 1.50 (1.07, 2.11) | 1.00 (0.93, 1.08) |
| **Volatile solvents** | 2.68 (1.92, 3.73) *** | 1.16 (1.07, 1.27) *** |
| **Alkyl nitrates** | 2.79 (0.80, 9.78) | 1.38 (1.10, 1.72) |
|  |  | |
|  | **Conduct disorder** | |
| **Nitrous oxide** | 2.51 (1.85, 3.40) *** | 1.14 (1.05, 1.26) |
| **Volatile solvents** | 3.14 (2.33, 4.25) *** | 1.24 (1.15, 1.34) *** |
| **Alkyl nitrates** | 2.06 (0.62, 6.79) | 1.13 (0.98, 1.30) |

^a^ All results estimated from imputed data.

^b^ Multivariable models adjusted for gender identity, ethnicity, free school meal entitlement, parent/caregiver unemployment, weekly cigarette smoking and consuming alcohol in the past 30 days.

^c^ Propensity score estimated using logistic regression with the teffects ipwra command in Stata v17.0. Covariates included were gender identity, ethnicity, free school meal entitlement, parent/caregiver unemployment, weekly cigarette smoking and consuming alcohol in the past 30 days.

^d^ Analytical n = 4813 as excludes those who responded that they preferred not to say or didn’t know whether they had hallucinated.
