# Supplementary material for: Association of volatile substance, nitrous oxide and alkyl nitrate use with mental health in UK adolescents
Source: Br J Psychiatry. Author manuscript; Available in PMC 2025 Jan 3. (PMC7617277; doi:10.1192/bjp.2024.128)
Supplement: Supplementary file 2 [file EMS198190-supplement-Supplementary_File__Supplementary_Tables__S1__S2__S3_.pdf]

Supplementary Online Content

Table S1. Number and percentage of missing data for each variable

Table S2. Odds ratio (95% confidence interval) for association between nitrous oxide, volatile solvents and alkyl nitrates and probable depressive disorder, generalised anxiety disorder, auditory hallucinations and conduct disorder in complete case sample (n = 3163)

Table S3. Odds ratio (95% confidence interval) for association between nitrous oxide, volatile solvents and alkyl nitrates with study outcomes, comparing multivariable adjustment and inverse probability weighting by a propensity score (n = 6612)

Table S1. Number and percentage of missing data for each variable

Characteristic	Missing	
	N	%
Exposure		
Nitrous oxide	156	2.3
Volatile solvents	154	2.3
Alkyl nitrates	179	2.7
Covariates		
Gender identity	68	1.0
Ethnicity	88	1.3
Free school meals entitlement	81	1.2
Parent(s) / carer(s) unemployment	606	9.1
Weekly smoking status	551	8.3
Alcohol consumed in past 30 days	760	11.4
Outcomes		
Probable depressive disorder	496	7.4
Probable anxiety disorder	602	9.0
Auditory hallucinations	292	4.4
Probable conduct disorder	631	9.5

Table S2. Odds ratio (95% confidence interval) for association between nitrous oxide, volatile solvents and alkyl nitrates with study outcomes in the complete case sample (n = 3163) [a]

Exposure variable	Unadjusted	Adjustment for other inhalants	Additionally adjusted for sociodemographic factors [a]	Adjusted for tobacco and alcohol use [c]
		**Probable depressive disorder**		
**Nitrous oxide**	4.37 (3.04, 6.28)	2.96 (1.99, 4.40)	3.22 (2.13, 4.87)	2.47 (1.61, 3.79)
**Volatile solvents**	4.73 (3.35, 6.68)	3.51 (2.42, 5.08)	3.11 (2.10, 4.62)	1.94 (1.29, 2.94)
**Alkyl nitrates**	4.09 (1.29, 12.96)	0.96 (0.27, 3.38)	0.88 (0.23, 3.39)	0.44 (0.11, 1.76)
		**Probable generalized anxiety disorder**		
**Nitrous oxide**	3.67 (2.54, 5.31)	2.40 (1.60, 3.61)	2.49 (1.63, 3.82)	2.02 (1.31, 3.11)
**Volatile solvents**	4.24 (3.00, 5.99)	3.17 (2.18, 4.61)	2.78 (1.87, 4.14)	1.92 (1.26, 2.90)
**Alkyl nitrates**	6.48 (2.01, 20.88)	1.93 (0.55, 6.78)	1.86 (0.48, 7.18)	1.25 (0.32, 4.86)
		**Auditory hallucination**		
**Nitrous oxide**	3.74 (2.59, 5.41)	2.03 (1.33, 3.09)	1.98 (1.28, 3.02)	1.57 (1.02, 2.43)
**Volatile solvents**	6.35 (4.49, 8.97)	4.89 (3.37, 7.08)	4.32 (2.95, 6.33)	2.95 (1.98, 4.39)
**Alkyl nitrates**	15.39 (4.09, 57.90)	4.46 (1.06, 18.73)	4.02 (0.91, 17.75)	2.56 (0.58, 11.24)
		**Conduct disorder**		
**Nitrous oxide**	5.21 (3.62, 7.49)	2.94 (1.96, 4.41)	2.94 (1.95, 4.43)	2.05 (1.32, 3.17)
**Volatile solvents**	7.45 (5.24, 10.58)	5.27 (3.63, 7.66)	5.06 (3.46, 7.39)	2.72 (1.81, 4.09)
**Alkyl nitrates**	48.45 (6.17, 380.48)	14.79 (1.65, 132.40)	15.52 (1.69, 142.48)	5.88 (0.64, 54.35)

[a] Multivariable model adjustment is incremental.

[b] Sociodemographic factors comprised: gender identity, ethnicity, free school meal entitlement and living with an employed parent.

[c] Tobacco use assessed as weekly cigarette smoking and alcohol assessed as consuming alcohol in the past 30 days.

[d] Analytical n = 4813 as excludes students who responded that they preferred not to say or didn't know whether they had hallucinated

Table S3. Odds ratio (95% confidence interval) for association between nitrous oxide, volatile solvents and alkyl nitrates with study outcomes, comparing multivariable adjustment and inverse probability weighting by a propensity score (n = 6672) [a]

	Multivariable adjustment [b]	Inverse probability
**Probable depressive disorder**		
**Nitrous oxide**	1.77 (1.33, 2.38) ***	1.03 (0.95, 1.12)
**Volatile solvents**	2.55 (1.90, 3.42) ***	1.15 (1.06, 1.25) ***
**Alkyl nitrates**	0.48 (0.18, 1.34)	0.88 (0.70, 1.10)
**Probable generalized anxiety disorder**		
**Nitrous oxide**	1.59 (1.19, 2.52)	1.01 (0.93, 1.10)
**Volatile solvents**	1.83 (1.37, 2.45) ***	1.08 (1.00, 1.16)
**Alkyl nitrates**	2.10 (0.74, 5.90)	1.21 (0.96, 1.51)
**Auditory hallucinations** [d]		
**Nitrous oxide**	1.50 (1.07, 2.11)	1.00 (0.93, 1.08)
**Volatile solvents**	2.68 (1.92, 3.73) ***	1.16 (1.07, 1.27) ***
**Alkyl nitrates**	2.79 (0.80, 9.78)	1.38 (1.10, 1.72)
**Conduct disorder**		
**Nitrous oxide**	2.51 (1.85, 3.40) ***	1.14 (1.05, 1.26)
**Volatile solvents**	3.14 (2.33, 4.25) ***	1.24 (1.15, 1.34) ***
**Alkyl nitrates**	2.06 (0.62, 6.79)	1.13 (0.98, 1.30)

[a] All results estimated from imputed data.

[b] Multivariable models adjusted for gender identity, ethnicity, free school meal entitlement, parent/caregiver unemployment, weekly cigarette smoking and consuming alcohol in the past 30 days.

[c] Propensity score estimated using logistic regression with the teffects ipwra command in Stata v17.0. Covariates included were gender identity, ethnicity, free school meal entitlement, parent/caregiver unemployment, weekly cigarette smoking and consuming alcohol in the past 30 days.

[d] Analytical n = 4813 as excludes those who responded that they preferred not to say or didn’t know whether they had hallucinated.
